# Preparation of Natural Tooth for Crown*This extract is taken from a paper read before the New York Second District Dental Society, and is published in full in The Dental Items of Interest for May, 1917.

**Published:** 1917-08

**Authors:** F. H. Orton


### PREPARATION OF NATURAL TOOTH FOR CROWN *

BY F. H. ORTON, D.D.S.

The removal and replacement of the cervical third of the enamel is the distinguishing requirement of the so-called ''banded'' crown. I say ''so-called'' advisedly, as I believe that the term ''band,'' used to designate a reproduction of the cervical enamel, is a misnomer. It is not an accurate architectural expression, symbolizing, as it does, a shape utterly unlike the cervical enamel. The definition of a band given by the New Standard Dictionary is, ''a flat flexible strip of any material used for binding.'' While this definition might be said to resemble in some respects the cervical form given the average crown, it does not in any respect reproduce the form or the function of the cervical enamel. I have looked in vain for some concrete term which would reflect in the mind an image of the form and function of this part of the enamel, and the nearest term I can find is the inadequate one of ''beveled ferrule.'' I realize that this term is only approximately correct, but it nevertheless implies a closer adaptation than does the term ''band,'' which seems to suggest something which lies over and on the outside of the root and resembles the fit of a barrel hoop, an idea far from the ideal of a banded crown. In the making, then, of a ferrule crown, we are confronted by another factor in the production of favorable conditions, *i. e.*, the principle that an exact duplicate of the gingival contour of the enamel, normally covered by the free gum margin, is of the first importance; for the success of the ferrule crown depends upon maintaining, not only a healthy gum margin, but also the integrity of the alveolar process, and this is unquestionably promoted by such contour. The effect of

* This extract is taken from a paper read before the New York Second District Dental Society, and is published in full in The Dental Items of Interest for May, 1917.

persistent local irritation upon these tissues is too well known to need comment. While the inflamed and congested condition of the gum margin found around the average banded crown may be due, in a large percentage of cases, to improper root preparation and faulty adaptation of the band, I believe that in those cases where the root preparation has been made in accordance with the prevailing practice, and where the band has been perfectly adapted around the entire gingival circumference, there would still be a failure to reproduce normal conditions, as a single band can seldom restore the gingival contour. It is a most difficult matter, indeed, to restore this essential feature of the tooth's anatomy with a flat band. As a matter of fact, the steps in the usual method, of cutting a piece of gold plate to measurement, bending to circular form, soldering, conforming to the shape of the root, trimming, and forcing to position, sound very simple, but they by no means constitute the only difficulties we have to contend with. We are confronted with the task of contouring the mesial and distal sides of the band to the marble-like contact, and of properly shaping the buccal and lingual surfaces without distorting the gingival adaptation. If gold were plastic like wax, it might be done; but it is not. The difficulties in the way of a practical solution are no doubt the chief reasons for neglecting these essential details.

In attempting to reproduce the gingival enamel, we should duplicate the form best suited to the maintenance of the health of the surrounding tissue. For instance, teeth possessing a pronounced gingival ridge, known as "bell crown teeth," afford the best protection to the free gum margin.,

When the enamel is removed and the root is prepared for the reception of the ferrule, a space is left representing the shape of the removed enamel. If the ferrule does not exactly duplicate the enamel, *i. e.*, if it does not exactly fit this space, an unfavorable condition must be produced; in other words, the gum should hug the crown as closely as it did the natural tooth.

Why study anatomy if we are not going to use it? Idle knowledge is as worthless as ignorance. The exhaustive treatment of the surface anatomy of the teeth by the several textbooks on the subject leaves no excuse for the conventionalized crowns in common use, as you will see for yourselves.

The preparation of the tooth for the reception of the ferrule crown introduces new problems, and makes new and searching demands upon our knowledge. We are confronted with the necessity for understanding the relation of the enamel and dentin to the contour of the tooth; and especially is it important to know the form of the enamel as it approaches the cementum; and yet, it is undeniable that the part of dental anatomy which has a special bearing upon crown and bridgework has been strangely neglected. This accounts for the fact that we have at the present time no systematized method of root preparation, such as we have, for example, for cavity preparation. The literature on the banded crown usually dismisses this important part of the operation with the brief caution to make the axial walls of the tooth parallel. It may be that the character of this work is such that it does not lend itself readily to the written word, for the operation must be visualized to be valuable. Individual instruction, such as can be given by means of clinical demonstration to small groups, is perhaps the ideal method for this part of our work; but even here a full appreciation of method depends upon a perception of the fundamental principles involved. Personally I see no reason why the principles which should guide this operation may not be as clearly presented to a larger assemblage as those governing cavity preparation.

Another parallel between these two problems is, that not until we applied our knowledge of anatomy to cavity preparation were the principles which govern the operation laid down and adopted, and there is no doubt of the fact that we are dependent upon our knowledge of anatomy to an even greater degree in the problem before us today.

When we stop to consider the firmness of the attachment between the enamel and the dentin, together with the fact that a certain part of the field of operation is concealed from view by a very delicate membrane which will usually react unfavorably to the slightest injury, you will all acknowledge that this work can not be performed in a perfunctory manner. Other things being equal, it is certain that he who depends upon a sense of touch alone in this work will do more damage to the surrounding tissues than he who is aided as well by a clear knowledge of the character and contour of the structure upon which he is working. When we are called upon to scale this enamel in our root preparation, our instrumentation often shows, from the laceration of the gum tissue, that we have failed to apply our knowledge of the anatomy of that portion which lies beneath the free margin of the gum tissue, and I think that one of the reasons for this is that we do not determine accurately the extent to which this gum cuff, or free gum margin, extends from the gingival line over the enamel of the crown. From the results which we often see, we are led to believe that the border of the free margin was mistaken for the gingival line. On this point let me quote from Dr. Damon, the dental anatomist of the University of Minnesota.

''Judging from a number of measurements taken,'' he says, ''I am of the opinion that we have a free margin which varies from one and a half mm. to three mm. in extent, in an adult, under normal conditions; this being somewhat greater in proportion with younger patients.'' It is generally conceded that the ferrule should extend rootwise as far as the gingival line, and as the gingival line is usually the most constricted portion of the tooth, the tooth must be scaled at least as far as this constricted gingival line, if we are to have a ferrule in close contiguity around the entire gingival circumference. How are we to be certain when we have scaled the tooth sufficiently to obtain this necessary condition? Will the removal of the enamel be sufficient to give us parallel walls, or does the dentin make up part of the gingival contour?

Even the removal of the enamel in its entirety from the dentin, for a space upwards of two mm. underneath the gum tissue, without in any way injuring it or the peridental attachment, requires so high a degree of surgical skill, and is so laborious an operation, burdensome alike to the patient and to the operator, that we must be quite sure of its necessity before insisting upon it as a normal procedure. A description of the relative proportion of the tooth occupied by each tissue will, I think, be convincing upon this point. The contour of the tooth is made up almost entirely of the enamel, beginning at the gingival line with a feather edge, which gradually increases in thickness until the cutting edge or points of the cusps are reached, at which point we find the thickest enamel. This enamel will be found thicker underneath any ridges or elevations on the various surfaces, the thickening formed by the increased convexity of the outer surface. The dentoenamel junction or axial walls of the dentin pass toward the occlusal with little or no convexity, and with only a slight inclination toward the axial lines of the tooth. The exception to this rule will be found in those teeth which go back to a very primitive type, in which extremely bell-shaped teeth the axial walls of the dentin on the mesial and distal surfaces are not inclined toward each other, but are almost parallel.

If this anatomical description is accurate, we see that by removing the enamel in its entirety, we have by that operation alone produced an almost ideal root preparation for the reception of our ferrule.

Now, the outline form of the dentoenamel junction is practically the same as the outline form of the dentin at the gingival line. We may easily verify this by using an extracted tooth; obtain the shape at the gingival by fitting a brass ligature wire around it at this point, and then grind down the occlusal until the free margin of the gum is reached. Now place the outline form of the gingival obtained from the wire ligature over the dentoenamel junction at the free margin, and it will be found to be almost identical. If we are to obtain perfect adaptation of the ferrule at the gingival, therefore, the removal of all the enamel at this point is obviously necessary, and if during the operation we bear in mind the points at which we may expect to find it the thickest, the procedure will be controlled by a more intelligent direction.

The enamel is thicker upon the mesial and distal than upon the buccal and lingual surfaces, and again thicker upon the distal than upon the mesial surface; and the fact that on the disto-lingual angle line we find the thickest enamel makes this the most difficult point to scale. This thickness we might expect, as the mesial root is larger and wider buccolingually than the distal root, which has as a rule only one canal, while the measurement of the periphery of the crown is the same buccolingually on the distal as upon the mesial surface. On the upper molars there is usually found a thickening of the enamel upon the mesiolingual and distolingual angle lines, which is especially difficult to scale. This thickening is probably due to the lessened mesiodistal diameter of the lingual root over that of the two buccal roots. Another point to be noted, in an upper first molar from which the distal convexity has been cut off, is that the enamel passes higher gingivally on the lingual than upon the buccal surface; and in a buccal view, at the point of approximal contact, it passes higher upon the distal than upon the mesial surface. This important anatomical point may usually serve as a guide for trimming and festooning the ferrule as well as in scaling the root.

I hope that in thus calling attention to the relation existing between the enamel, the dentin, and the contour of the tooth, the necessity for the removal of the enamel in its entirety has been so conclusively demonstrated as to furnish a basis for one fundamental principle in the construction of the banded crown.

If we adopt this as a standard from which a proper root preparation for a banded or ferrule crown is to be judged; and if we also adopt the exact reproduction of the surface anatomy of the natural teeth (especially those forms which are recognized as having a particular bearing upon the production of favorable conditions) as our standard of criticism for the finished crown, we shall at least have laid down the principles which should govern the practice of crown work, and will have put this work on a plane which will not make it inconsistent with the fundamental laws of prophylaxis.

